# Preclinical trial of noncontact anthropometric measurement using IR-UWB radar

**DOI:** 10.1038/s41598-022-12209-1

**Published:** 2022-05-17

**Authors:** Jinsup Kim, Won Hyuk Lee, Seung Hyun Kim, Jae Yoon Na, Young-Hyo Lim, Seok Hyun Cho, Sung Ho Cho, Hyun-Kyung Park

**Affiliations:** 1 Department of Pediatrics, Hanyang University College of Medicine, Seoul, 04763 Republic of Korea; 2 Department of Electronics and Computer Engineering, Hanyang University, Seoul, 04763 Republic of Korea; 3 Division of Cardiology, Department of Internal Medicine, Hanyang University College of Medicine, Seoul, 04763 Republic of Korea; 4 Department of Otorhinolaryngology, Hanyang University College of Medicine, Seoul, 04763 Republic of Korea

**Keywords:** Preclinical research, Electrical and electronic engineering

## Abstract

Anthropometric profiles are important indices for assessing medical conditions, including malnutrition, obesity, and growth disorders. Noncontact methods for estimating those parameters could have considerable value in many practical situations, such as the assessment of young, uncooperative infants or children and the prevention of infectious disease transmission. The purpose of this study was to investigate the feasibility of obtaining noncontact anthropometric measurements using the impulse-radio ultrawideband (IR-UWB) radar sensor technique. A total of 45 healthy adults were enrolled, and a convolutional neural network (CNN) algorithm was implemented to analyze data extracted from IR-UWB radar. The differences (root-mean-square error, RMSE) between values from the radar and bioelectrical impedance analysis (BIA) as a reference in the measurement of height, weight, and body mass index (BMI) were 2.78, 5.31, and 2.25, respectively; predicted data from the radar highly agreed with those from the BIA. The intraclass correlation coefficients (ICCs) were 0.93, 0.94, and 0.83. In conclusion, IR-UWB radar can provide accurate estimates of anthropometric parameters in a noncontact manner; this study is the first to support the radar sensor as an applicable method in clinical situations.

## Introduction

Assessment of anthropometric profiles (height, weight, body mass index [BMI], body circumference, and skin-fold thickness) plays an important role in the evaluation of various medical conditions^1,2^. In particular, a person’s height and weight are the core elements of anthropometry and indicators of his or her physical aspects and are crucial in monitoring the state of the disease or assessing growth and development according to nutritional conditions in infants and children^2,3^. Changes in weight are important information for monitoring the health of infants and children and evaluating their growth and development. During medical emergencies or serious conditions in the intensive care unit, accurately measuring the weight of patients can be difficult due to difficulty in moving patients with mental/physical disabilities; one solution to this type of problem may lie in noncontact measurement^4–6^, which has demonstrated considerable value in the forensic, online business, and virtual environment domains. The recent global COVID-19 pandemic has raised issues related to the transmission of infectious diseases and the urgent need for the development of noncontact technologies.

In general, height and weight are measured indoors using traditional methods involving physical contact, such as a scale. Several studies have employed various techniques to measure these two values using noncontact, accurate, and convenient approaches, including Kinect sensors, 3D structured light sensors, ultrasonic sensors, estimations from images or video, microdoppler radar and so on^4,7–13^. Kinect sensors have also been used to accurately measure parameters of the human body with large-scale motion under the assumption that the individual was wearing clothes^7^. However, thus far, these methods have not overcome the technical limitations of the sensors^7^. Ultrasonic sensors are cost-effective devices for measuring the size of the human body at distances up to 300 cm despite variations in body shape^9^. The major difficulty in noncontact weight measurement is the characterization of the main anatomical and compartmental components. Spatiotemporal analysis for mining information across posture variations is needed to measure body parameters.

Impulse-radio ultrawideband (IR-UWB) radar is a high-precision electromagnetic technique that recognizes the motion of an object at a distance and, unlike video images, lacks privacy issues. This radar has various advantages over other techniques in medical applications, such as its contactless/wireless and license-free nature, ease of application, inexpensiveness, high data-processing rate, low exposure risk for the human body, and daily convenience in and out of the hospital. Recently, we introduced a signal model for IR-UWB radar and an algorithm for noncontact, continuous measurement of tiny movements and vital signs (respiratory and heart rates) of the human body, even for small, premature infants^6,14–18^. The results of these studies demonstrated the applicability of IR-UWB radar in the assessment of medical status using distance information. The algorithm for calculating body parameters uses a convolutional neural network (CNN) to analyze patterns extracted from the images from IR-UWB radar. This noncontact sensing technology can measure height and weight without any limitation to the value range and can be implemented with the Internet of Things (IoT) for remote health monitoring in digital healthcare markets, overcoming the issues of contact and infection in the COVID-19 pandemic.

To the best of our knowledge, this work is the first attempt to estimate anthropometric parameters by exploiting a novel, noncontact IR-UWB radar sensor. The purposes of this study are to propose a unique algorithm for measuring these parameters with radar, to implement a CNN with optimized layers to achieve an algorithm with maximum accuracy, and to evaluate the accuracy of the algorithm and compare it to that of traditional methods.

## Materials and methods

### Subjects

To acquire IR-UWB radar data, we recruited a total of 45 adults (19 men and 26 women) over the age of 20 from Hanyang University Hospital who participated voluntarily from January to April 2021. This study was approved by the institutional review board of Hanyang University Medical Center (No. 202101-015). All methods were performed in accordance with standard human research ethics guidelines (Declaration of Helsinki) and regulations. Written informed consent was provided by all patients before they were enrolled in the study. Additionally, no participants had a history of mental illness, trauma or diseases of the nervous system or musculoskeletal system.

### Experimental setup and data collection

All experiments for data collection were conducted in the multidisciplinary room of the Inclusive Clinic for Developmental Disorders at Hanyang University Hospital, Seoul, Korea.

#### Reference methods

**Figure 1:**
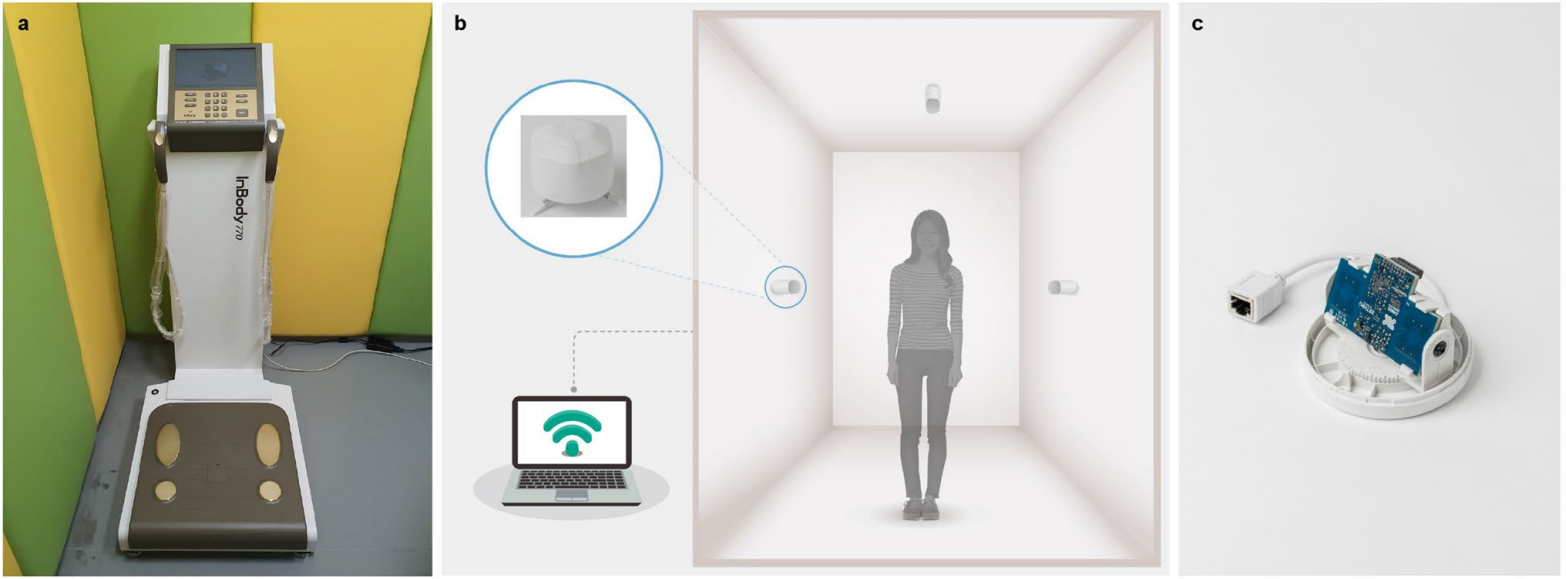
Overall experimental environment for data collection. (**a**) BIA (InBody 720), (**b**) radar sensor settings, and (**c**) IR-UWB radar chip (XK350-120 W0) covered with a plastic cap.

Height was measured with a calibrated, wall-mounted stadiometer, and bioelectrical impedance analysis (BIA) was conducted using InBody 720 (Biospace Co., Seoul, Korea; Fig. 1a) to measure weight (kg), BMI (kg/$\hbox {m}^2$), muscle mass percentage (%), skeletal muscle mass percentage (%), body fat mass percentage (%), and body water mass percentage (%). BIA is a useful and well-studied technique for measuring body fat relative to lean mass^19^. Weight was also measured by a digital scale to the nearest 50 g, and height was evaluated to the nearest 0.1 cm.

#### IR-UWB radar sensor

Next, the participant stood motionless for 5 seconds in the middle of the room installed with three commercially available IR-UWB radar sensors (XK350-12 W0, Xandar Kardian, Delaware, USA) for data collection; each radar sensor was encapsulated with a white plastic cover (Fig. 1b). The room measured 3 m $\times$ 3 m $\times$ 2.5 m wide; one of the three IR-UWB radar sensors was installed in the center of the ceiling of the room, and the other two were installed on opposite walls 1 m above the floor (Fig. 1c). The center frequency of the radar sensors used for data collection was 8.748 GHz, and the received signal was sampled at 23.328 GS/s at the receiver. The bandwidth was approximately 1.5 GHz, and the transmitted radiation power was 68.85 mW; this transmission power complied with FCC mask indoor standards, and the signals transmitted from each radar sensor did not interfere with each other. The distance resolution was 6.4 mm, which was suitable for capturing minute body movements. The signal transmitted from the radar sensor reached the test participant’s body and was reflected back to the receiver, which then transmitted the sampled signals to the PC through USB at a rate of 20 times per second. All radar signals transferred to the PC were processed through MATLAB (MathWorks, MA, USA). All participants were tested 8 times to collect IR-UWB radar data.

### Statistical analysis

Experimental results data are presented in scatter plots in terms of the root-mean-square error (RMSE), mean absolute error (MAE), intraclass correlation coefficient (ICC) and Pearson correlation coefficient ($r^2$) values with the reference method due to the characteristics of the data. A scatter plot was used to compare the concordance and correlation between the measured values and the estimated values. Statistical analysis was performed using MATLAB version 2021a.

## Algorithm for measuring anthropometric parameters

### System overview

**Figure 2:**
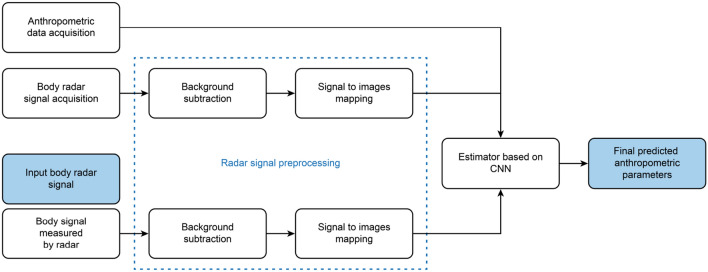
Proposed method for anthropometric parameter estimation using a convolutional neural network.

The block diagram for the proposed method is shown in Fig. 2. Human body signals collected from three radars were input into the CNN following preprocessing and image mapping and trained through the CNN together with the measured anthropometric parameters. The dataset for verifying the trained CNN also underwent the same signal processing, and an anthropometric parameter prediction was generated through the trained CNN.

### Radar signal preprocessing

The impulse signal transmitted from the radar sensor is reflected off not only the participant’s body but also objects in the experimental environment. The signal $x_{i,n}[k]$ received from the *i*-th radar sensor at the *n*-th time can be modeled as follows.

1 $$\begin{aligned} x_{i,n}[k] = \sum _{m=1}^{N_{path}} A_{m,i,n}S[k-\tau _{m,i,n}]+N[k] \end{aligned}$$

where *k* is a distance index and has a natural number value from 0 to the length of the radar signal. *A* and $\tau$ are the delay and scaling factors created for the *m*-paths of the transmitted impulse signal returning to the receiver, respectively. *N*[*k*] is the noise component in the experimental environment^20^. Since the received signal contains not only the signal for the target but also that for the components of the experimental environment, it is not suitable for immediate use in anthropometric parameter measurement; these clutter signals have a larger magnitude than the target signal and thus are difficult to distinguish. Instead, we remove the clutter signal from the received signal using a background subtraction algorithm, leaving only the target signal and the experimental participants’ signals to be extracted^21^.

In the background subtraction algorithm, clutter signal $C_{i,n}[k]$ is continuously updated over time, and signal $y_{i,n}[k]$, in which the background signal is removed, is obtained by subtracting the clutter signal $C_{i,n}[k]$ from the original signal $x_{i,n}[k]$. The alpha value can take a value between 0 and 1; in this study, it is set to 0.95.

2 $$\begin{aligned} \begin{aligned} y_{i,n}[k]&= x_{i,n}[k] - C_{i,n}[k], \\ C_{i,n}[k]&= \alpha {}C_{i,n-1}[k] + (1- \alpha )x_{i,n}[k] \end{aligned} \end{aligned}$$

#### Height estimation using radar signal processing

Height can be obtained through a preprocessed signal extracted only from the radar sensor installed on the ceiling. Since we already know the distance between the radar sensor installed in the ceiling and the ground, if we can accurately measure the distance between the sensor and the top of the head when the participant is standing in the experiment room, we can calculate his or her height. To achieve high measurement accuracy, we use the constant false alarm rate (CFAR) algorithm on the given signal $y_{i,n}[k]$ to measure the height^22^. This algorithm is used to detect a target; one such algorithm is the cell-average constant false alarm rate (CA-CFAR), which implements a window of a certain size in one frame of data received from the sensor. First, a threshold is generated according to the CA-CFAR algorithm from signals collected in an experimental environment without a target. A target can then be detected by comparing the generated threshold with the signal received when a target is present. This threshold is created by setting the probability of a false alarm; when there is no target, as many as $N_c$ received signals are taken as absolute values after applying the Hilbert transform.

3 $$\begin{aligned} \begin{aligned} H_{i}[k] =[h_{i,0}[k], h_{i,1}[k], h_{i,2}[k], \ldots , h_{i,N_c}[k]]^T \end{aligned} \end{aligned}$$

where $h_{i,n}[k]$ is the signal resulting from application of the Hilbert transform and absolute value function to the background-subtracted signal $y_{i,n}[k]$ when no one is in the experimental environment, and $H_{i}[k]$ is an array stacked with up to $N_c$ of these signals to determine threshold $T_{i}[k]$. In Eq. (4), the mean value of the signal $H_{i}[k]$ collected in the empty experimental environment is $\sigma _{i}[k]$, $\mu _{i}[k]$ is the standard deviation of $H_{i}[k]$, and $\beta$ is a constant used for adjusting the false alarm rate. The environment-adapted threshold $T_{i}[k]$ is used to obtain the distance from the sensor installed on the ceiling to the tip of the head given signal $y_{i,n}[k]$ collected when a person is present.

4 $$\begin{aligned} \begin{aligned} T_{i}[k] =\beta \sigma _{i}[k] + \mu _{i}[k] \end{aligned} \end{aligned}$$

### Proposed CNN architecture

**Figure 3:**
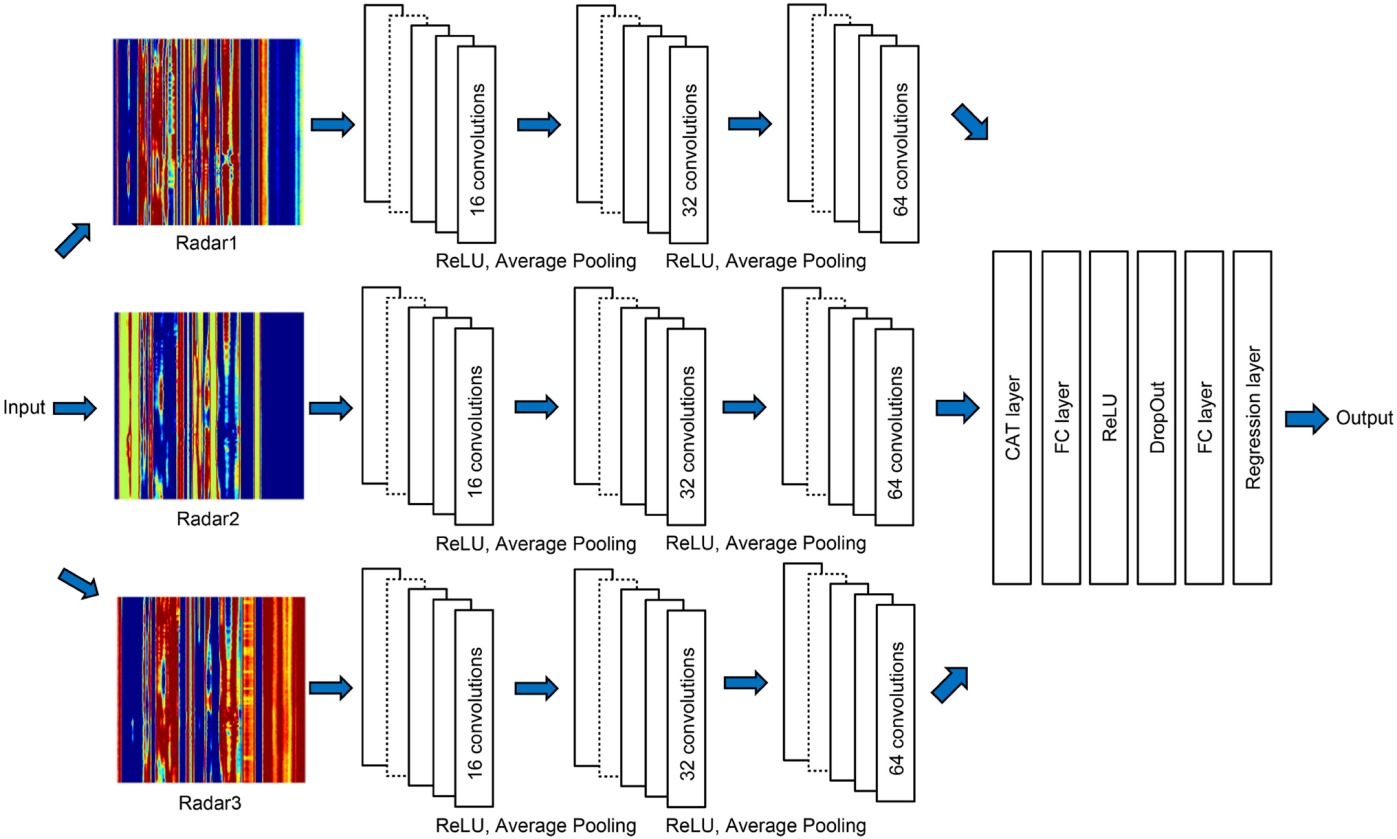
Architecture of the implemented convolutional neural network.

Aside from height, the other anthropometric parameters are difficult to obtain through signal processing methods. Most of those measured through BIA are values related to volume or mass, and most of the IR-UWB radar signals are reflected from human skin and cannot penetrate the human body due to the frequency band used and other characteristics of the transmitted impulse signal^23^. In other words, the mechanism of the radar system used in this study is not designed to obtain bioelectrical impedance or to directly measure anthropometric parameters using radio waves. Given these characteristics, the received signal is one-dimensional data whose signal strength is related to distance; it is thus difficult to estimate the indicators related to the human body through a single radar signal. Therefore, we attempt to use all signals received from the three radar sensors and propose a method for predicting the anthropometric parameters through the maximum likelihood method by imaging each subject’s radar signal and applying it to the CNN. After data preprocessing through the background subtraction algorithm, the training data were generated from radar signals with a size of 100 $\times$ 400. Since the FPS of the radar used is 20 and the range resolution is 6.4 mm, the radar signal used for training is collected for 5 s at distances up to approximately 2.4 m. Then, the data from the 3 radar sensors are stacked and rescaled from 0 to 255, which matches the 8-bit color depth range. The proposed algorithm uses color images as the input data; therefore, single-channel images are converted to RGB channel jpeg images using the conversion function with a given color map. Finally, the image is resized to (227,227,3), where 3 is the number of RGB color channels. Sample training images are shown in Fig. 3. The accuracy in estimating an anthropometric parameter depends on the quality of the image generated from the radar signal used as the CNN input. From the preprocessed radar signal, we remove the signal corresponding to the clutter component in the experimental environment and use only the signal corresponding to the target. These signals all vary depending on the participant in the experiment. This is because each participant in the experiment has a different area to reflect the radar’s transmission signal. The RCS (Radar Cross Section) is a measurement parameter of how many electromagnetic waves reflect in the experimental participant. It is difficult to express mathematically because the shape of the human body is irregular, but it is obvious that people with relatively large volumes and large areas have a higher RCS value than those who do not. The signal strength of a person with a high RCS would be stronger than that of one without, and we assumed that this signal would contain relevant information about the volume and area of the participant in the experiment.

In this way, features can be extracted through a signal processing method using radar signals without the need for pre-extracted features. Although various types of CNN structures can be applied to radar signals, it is more suitable to predict anthropometric parameters by applying each radar signal to an independent convolution layer rather than attaching all radar signals to one image and using it as input. The datasets created by each radar sensor have characteristics of human signals that depend on the location where it is installed, and since three sensors are used, a late-fusion method, rather than feature-level fusion, is used for the CNN structure, given the multimodality^24,25^.

Traditional and widely used CNN structures are advantageous for classifying images used as inputs; however, the amount of data collected in our study is not considered to be an input suitable to those public algorithms. Instead, we have proposed our own CNN structures to reduce errors in the anthropometric parameters with the BIA results through the proposed CNN structures, which were recently developed by our research team and have demonstrated effectiveness in image processing^26–28^. In our proposed CNN structure, the input image is composed of three preprocessed radar signals. The input images generated from each radar sensor are split, used to train the network, and concatenated in the CAT layer. The size of the filter used in the 2D convolution layer is 3 $\times$ 3, and the rectified linear unit (ReLU) layer is used as the activation function. In general, the ReLU layer is added after the convolution layer in the CNN structure using images. The operation order of each layer is convolution, ReLU, and average pooling. The final feature is created in the last fully connected layer, and the anthropometric parameters are estimated through a regression layer. Table 1 shows the details of the design of the proposed CNN architecture.

**Table 1:** Hyperparameter values of the proposed convolutional neural network.

Hyperparameter	Value
Number of hidden layers in CNN	3
Convolution filter size	3
Learning rate	0.001
Minibatch size	8
Number of epochs	60

## Results

### Baseline subject data

A total of 45 subjects (19 males, 26 females) were enrolled, and their characteristics are shown in Table 2. The median (interquartile range) height measured by the stadiometer was 164.00 cm (160.95–172.82). The median (interquartile range) weight and BMI obtained from BIA were 62.20 kg (55.30–74.62) and 22.80 (20.57-26.00), respectively. The median percentages of skeletal muscle mass, muscle mass, body water, and body fat were 22.70 % (20.40–31.42), 39.20 % (35.80–52.52), 30.60 % (27.95–40.85), and 17.90 % (15.30–22.55), respectively.

**Table 2:** Baseline subject characteristics.

Demographics	*N*=45
Age, years	33 (26.75–45.50)
Male	19 (42.22%)
Height, cm	164.00 (160.95–172.82)
Weight, kg	62.20 (55.30–74.62)
Body mass index, kg/m$^2$	22.80 (20.57–26.00)
Muscle mass, %	39.20 (35.80–52.52)
Skeletal muscle mass, %	22.70 (20.40–31.42)
Body water, %	30.60 (27.95–40.85)
Body fat, %	17.90 (15.30–22.55)

### Results of preprocessing the radar signal

**Figure 4:**
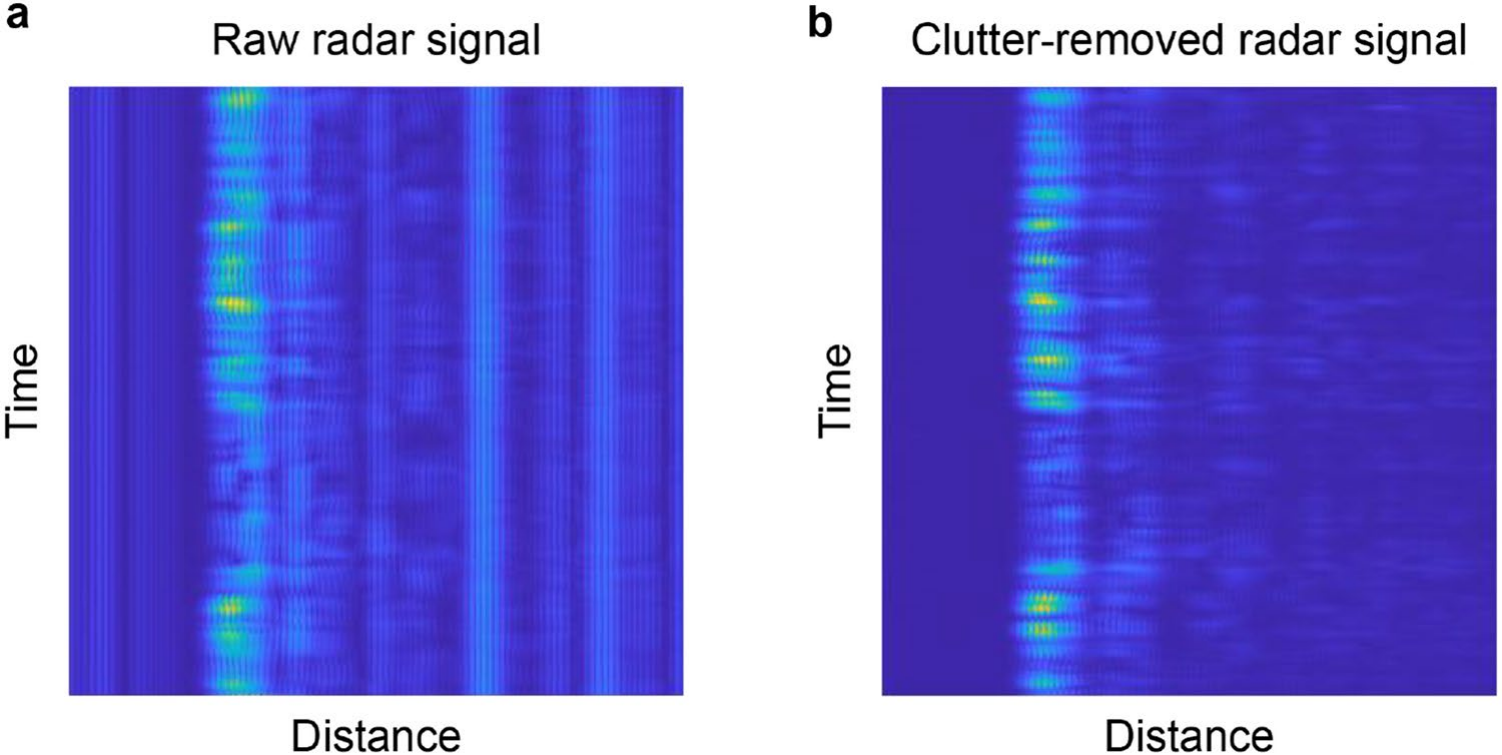
Background subtraction algorithm. (**a**) Signal before background subtraction. (**b**) Signal after background subtraction.

Figure 4 shows the implementation of the background subtraction algorithm presented in “Radar signal preprocessing”; all signals received from the 3 radar sensors were subjected to the proposed algorithm, which involved removing the signals corresponding to the background detected in the raw radar signals. Even if the subject was able to stand still in the environment for 5 s, the signal to the target could be acquired due to the generation of minute movements as well as those associated with breathing. Regardless of the experimental environment, the background signal can always be removed to produce a signal for training and validation so long as the installed positions of the sensors are consistent.

#### Height estimation by signal processing

**Figure 5:**
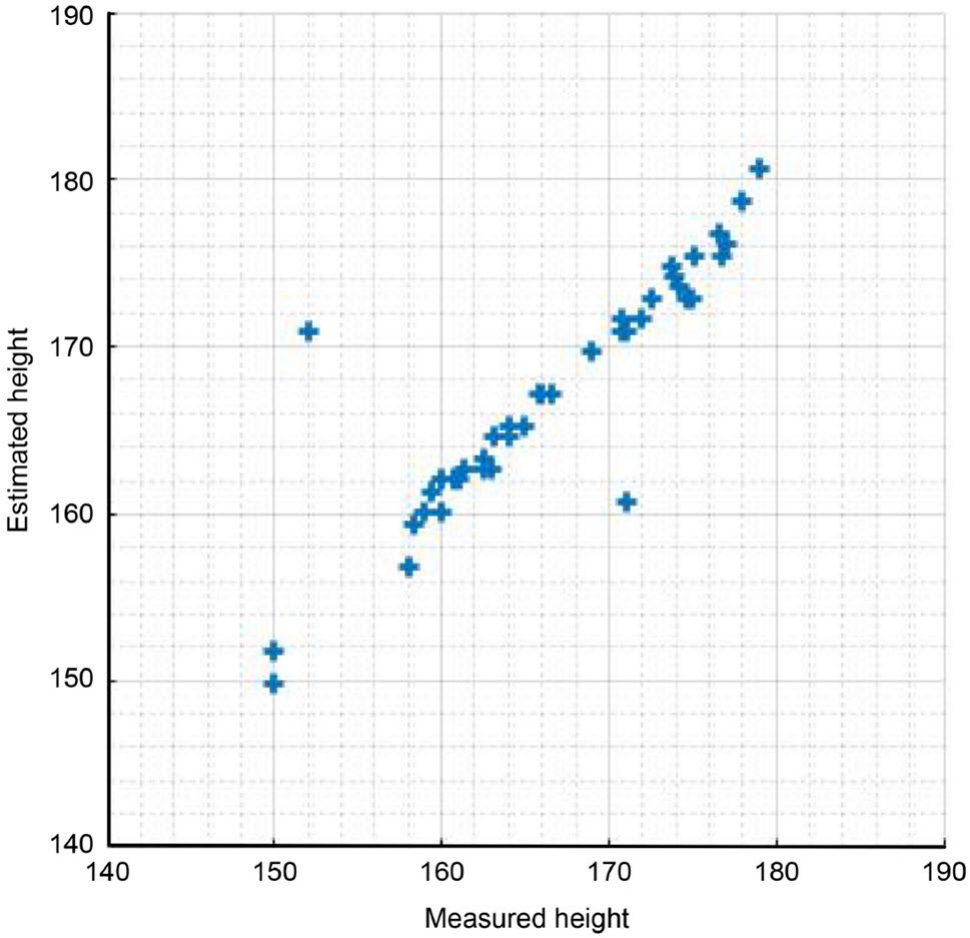
Comparison of estimated and measured height values.

To estimate the height through signal processing, one data point per person was used among the collected data. Of the three radar sensors installed in the experimental environment, only the data from the sensor installed on the ceiling were used. To obtain the threshold, data were collected from the experimental environment for 5 minutes, and the height was estimated by comparing the radar signal $y_{1,n}[k]$ obtained for each participant with the threshold $T_{1}[k]$. Figure 5 is a scatter plot of the height estimated through signal processing of the preprocessed radar signal; the MAE is 1.49, and the RMSE is 3.37. The ICC value between the estimated data and the measured data is 0.92, and the Pearson correlation coefficient $r^2$ is 0.85.

### Estimation results

**Figure 6:**
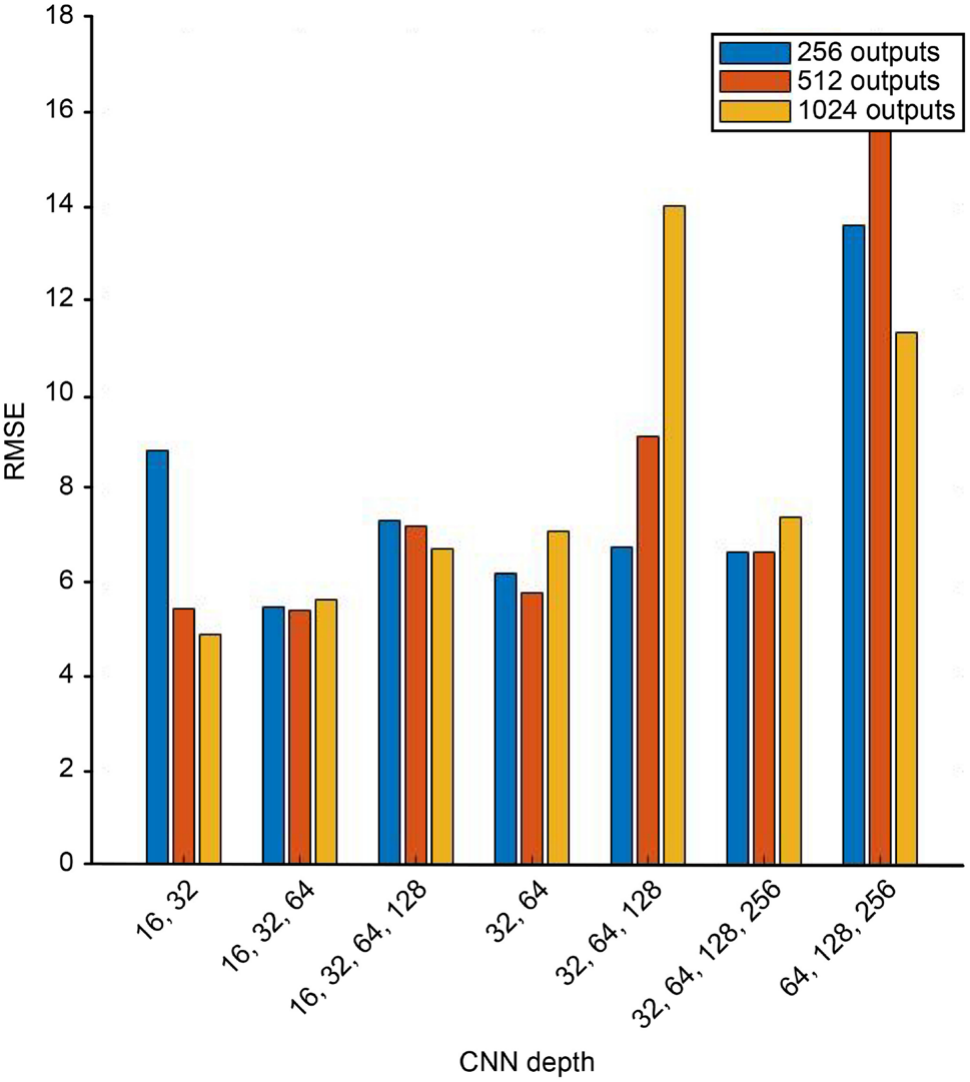
RMSE comparison for weight estimation.

To assess the accuracy of the proposed CNN architecture in weight estimation, we implemented different values for the CNN depth, the number of filters for the 2D convolution layer and the number of neurons in the fully connected layer. Of a total of 360 datasets collected from 45 people, 75% were randomly selected as the training set, and the remaining 25% were selected as the validation set. In Fig. 6, different colors represent different numbers of outputs of the fully connected layer used after the CAT layer: 256 in blue, 512 in red, and 1024 in yellow. When the number of hidden layers is 2, the number of filters for the 2D convolution layer is 16 or 32, and the output of the fully connected layer is 1024; the weight estimation has the smallest RMSE, 5.31. In this work, when the number of filters used in the 2D convolution layer increased excessively, the RMSE value also increased; similarly, when the depth of the network increased, the RMSE value also increased for 256 and 512 fully connected layers. For 1024 outputs of the fully connected layer and 32, 64, and 128 2D convolution layers, the RMSE values were greater than 7, the value obtained for 256 and 512 fully connected layer outputs.

**Figure 7:**
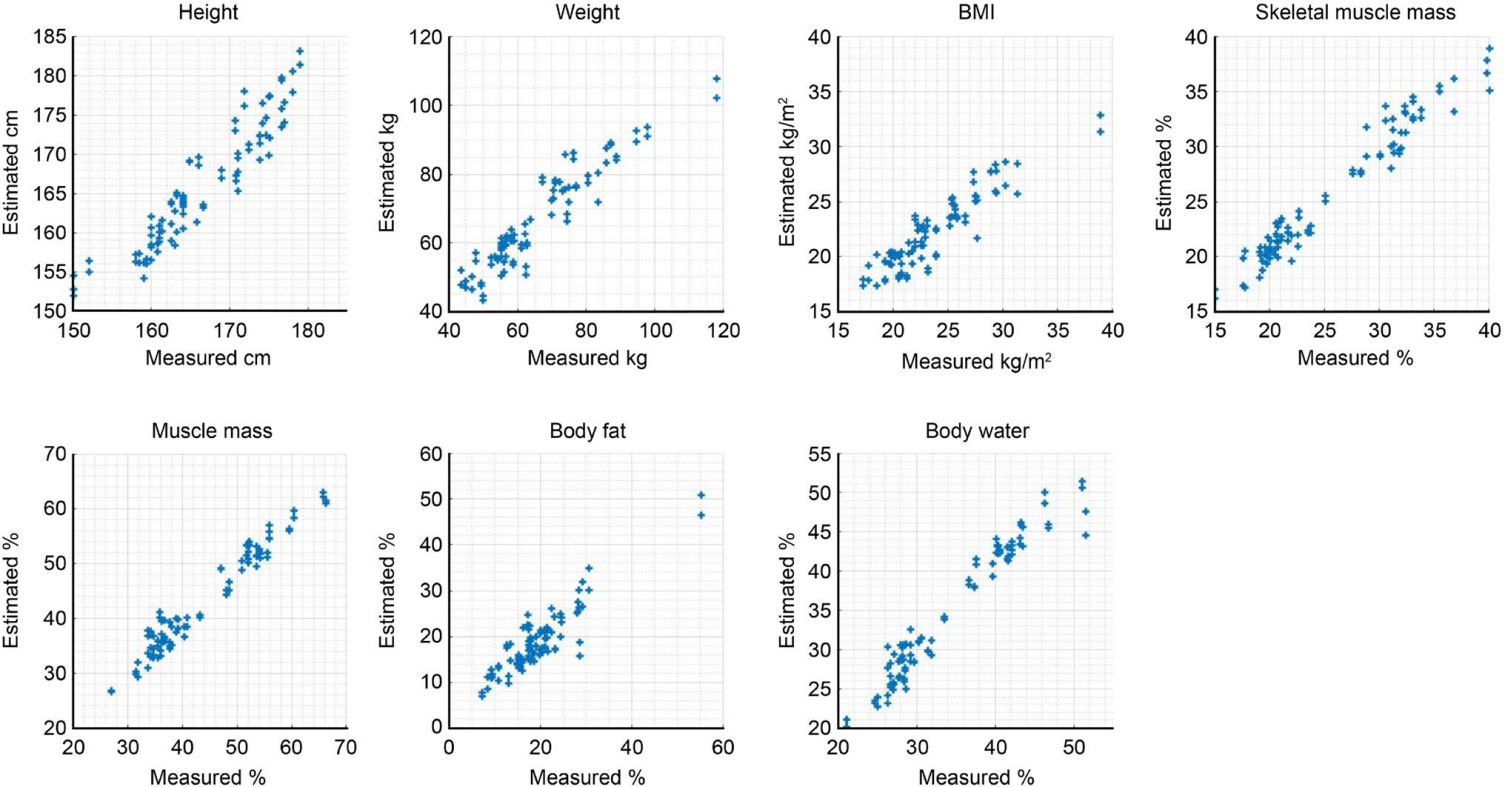
Comparison of estimation values and measured values of anthropometric parameters.

The networks were independently trained according to the seven measured anthropometric parameters, and all their structures had the same hyperparameter values. The diagrams in Fig. 7 illustrate the result of the proposed CNN architecture by plotting the measured and estimated values of the anthropometric parameters; all plots demonstrate positive correlations and linear distributions of the two sets of values. In Fig. 6, the error values of the heights of some participants are very large, but there was no such error value in the presented CNN-based results. These scatter plots show that all anthropometric parameters were sufficiently trained in the proposed structure.

**Figure 8:**
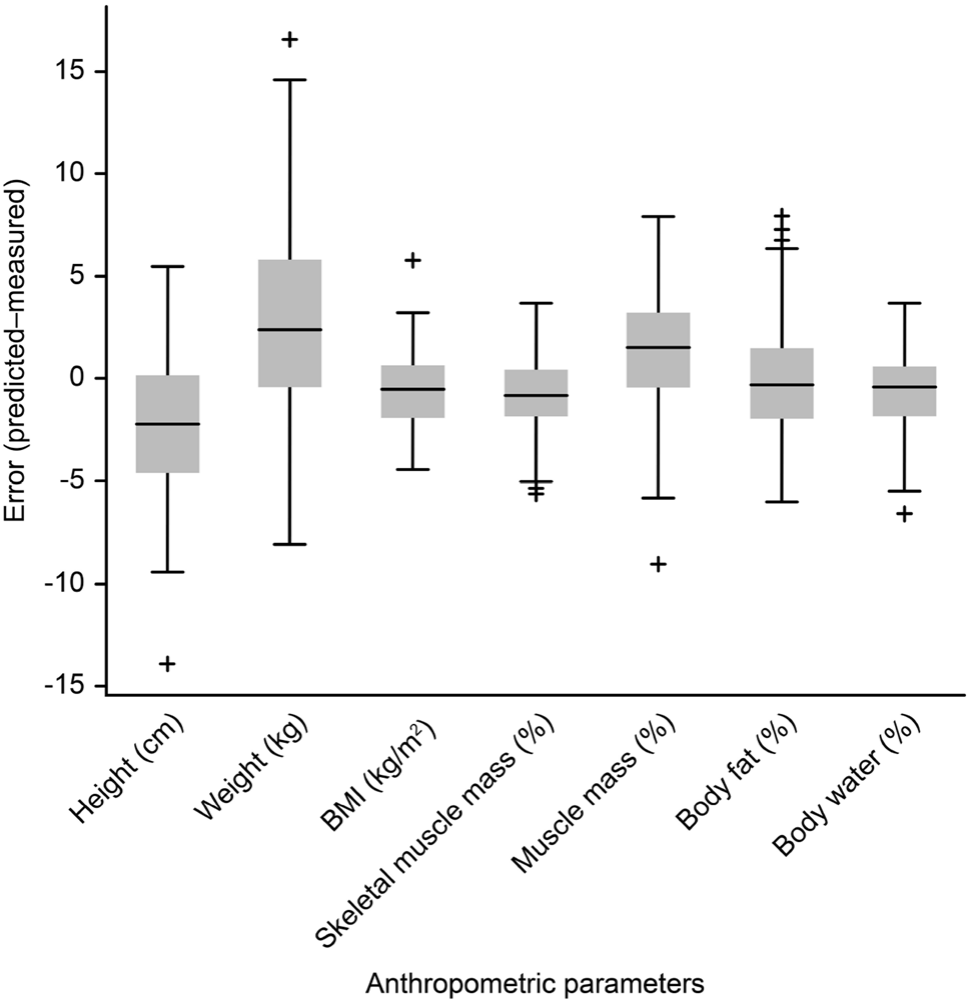
Box plot for estimation error.

The distribution of errors for the predicted values of each anthropometric parameter is shown in Fig. 8. For each box, the mark in the center represents the median, and the bottom and top edges of the box represent the 25th and 75th percentiles, respectively. Outlier ranges indicated by dotted lines are values that are at least 1.5 times the interquartile range from the bottom or top of the box, and data displayed outside the outlier range are shown in red. The median values for the errors of all indicators are distributed within 5, and some data for body fat are beyond the range of the marked outliers. Table 3 shows the results of the network trained for each anthropometric parameter in the proposed CNN architecture. The RMSE (5.31) and MAE (4.28) for weight were higher than those for the other parameters; these values indicate an error of less than 10% relative to an average weight of 62.00 kg, and the ICC value (0.96) indicates excellent agreement between the radar sensor and BIA. The ICC value of BMI (0.83) was lower than that of the other parameters; however, the absolute value of the error (RMSE 2.25, MAE 1.66) was within 10% of the average BMI (22.80 kg/$\hbox {m}^2$). The ICC values of the other parameters (> 0.90) and their low RMSE and MAE values also indicate excellent agreement between the radar sensor and BIA.

**Table 3:** Estimation results from the CNN.

Anthropometric parameter	RMSE	MAE	ICC	$r^2$
Height, cm	2.78	2.36	0.93	0.87
Weight, kg	5.31	4.28	0.94	0.88
Body mass index, kg/m$^2$	2.25	1.66	0.83	0.84
Skeletal muscle mass, (%)	1.48	1.16	0.97	0.95
Muscle mass, (%)	2.34	1.95	0.97	0.96
Body water, (%)	1.98	1.59	0.97	0.95
Body fat, (%)	3.36	2.53	0.90	0.82

## Discussion

In this study, our innovative, noncontact technology, IR-UWB radar, successfully measured anthropometric parameters with a high degree of accuracy comparable to that of BIA. Moreover, we demonstrated a state-of-the-art algorithm for simultaneously estimating the parameters using a machine learning scheme and a CNN architecture. The drawback of requiring multiple radar sensors remains a challenge that must be addressed in future work.

Anthropometric profiles are important for assessing obesity or other medical conditions that affect body weight or body shape. Obesity significantly increases the risk of cardiovascular disease, hypertension, diabetes mellitus, and many other diseases. The anthropometric profiles of infants and children must be measured to assess their nutritional status^2^. Moreover, BMI-for-age is commonly used as an index to identify the severity of obesity or malnutrition^2^. Additionally, anthropometric measurements can be used as a baseline for physical fitness in athletes and to measure the progress of fitness. A number of techniques have been used to assess body composition; for example, BIA is simple and easy to perform, but it requires the placement of electrodes on the body, and measurements should ideally be made with the subject at rest. Dual energy X-ray absorptiometry and computed tomography require expensive equipment and trained technicians and expose the subject to radiation^29–32^.

The 3D structure light sensor (Libra3D) has been shown to be an accurate approach to weight measurement relative to other conservative techniques. There are some limitations, however, due to its reliance on optical technologies alone: (1) larger measurement errors for very muscular, obese, or very thin patients; (2) the lack of cover with a blanket; and (3) unsuitability for patients who cannot be positioned with their back on the stretcher^4^. Recently, a noncontact ultrasonic sensor demonstrated good results with a low average error in measuring height and weight; this device is capable of overcoming one major drawback, weight change with altitude, of traditional weight measurements using a scale, but clothes should fit tightly to the body so that the volume estimation step can give a reasonable result^9^. Anthropometric measurement methods from images obtained from a camera demonstrated an acceptable level of precision feasible even in unconstrained images^10,11^.

Noncontact monitoring technologies play a vital role in the COVID-19 pandemic era; as it is important to minimize contact, such technologies can lower the risk of infection when measuring anthropomorphic profiles. Individual height and weight estimation through noncontact methods or images could have considerable value in surveillance/screening for school health and emergency situations, pedestrian traffic studies for urban planning, forensic science/intrusion detection, automated garment fitting in online stores, and autonomous driving. In particular, these techniques can be used appropriately in digital healthcare markets and the COVID-19 era of non-face-to-face business and virtual environments^10^. IR-UWB radar is an unobtrusive, cost-effective, easily applicable, low-risk, and contactless system that, in previous studies, we suggested as a new method for measuring vital signs in a noncontact manner. We also reported the accuracy of IR-UWB radar in monitoring heart rate and respiratory rate in adults, infants and patients under nocturnal polysomnography. Through these studies, we suggested that IR-UWB radar could accurately measure infant signals despite being of smaller amplitude and higher frequency than those of adults and is thus sufficiently clinically applicable^6,14,33–35^. We found that IR-UWB radar could measure anthropometric parameters without the use of physical contact and radiation, and we validated the accuracy measured by radar for the first time. In the measurement using the radar system, the radar can be easily installed alone, and results similar to BIA can be obtained by standing still at the measurement location for 5 s without contact with the subject.

We used three IR-UWB radar sensors for body data collection, transformed the preprocessed radar signal into an image, and then extracted the features using those images with the proposed CNN architecture. The training method of imaging radar signals and applying them as input to a CNN has been conducted in other studies^36^. Our results showed that IR-UWB radar could accurately measure anthropometric parameters in a noncontact manner and that the accuracy of the radar system was sufficiently high, suggesting its clinical applicability in situations such as medical emergencies. Since most drug doses used in emergency situations are based on weight, especially in children, when the patient’s weight cannot be measured, an accurate, rapid estimation of weight is important for administering the correct amount of medication and appropriate emergent intervention^37,38^. Several methods are commonly used for weight estimation, such as visual estimation, formulas and habitus modified systems; however, these methods bear the risk of estimation errors. Accuracy of at least PW10> 70% and PW20> 95% could be considered a reference standard (PW10; percentage of weight estimates within 10% of actual weight, PW20; percentage of weight estimates within 20% of actual weight)^39^. Even with Pediatric Advanced Weight Prediction in the Emergency Room (PAWPER) tape, the most accurate weight estimation device for children, the percentage of estimates within 10% of the actual weight is less than 80, and with the Lorenz method, the most accurate weight estimation device for adults, the percentage of estimates within 10% of the actual weight is less than 90^39–43^. Weight estimation using radar measured subjects’ weight within 10% of the weight measured using BIA, and the ICC value (0.96) indicates excellent agreement between the radar sensor and BIA. The results demonstrated that IR-UWB radar can feasibly measure patient weight when the weight cannot be measured directly. Additionally, it can be used as an indirect diagnostic basis for diseases that show rapid weight change. When severe hydration accompanied by weight loss of 10% or more appears in diabetic ketoacidosis or thyrotoxicosis, weight change beyond the margin of error suggested in this study may occur in adults.

Although the proposed CNN structure is efficient with the dataset used in this study, it cannot be said to be optimal. Because the number of datasets used in this study was not large, a network with a fairly deep structure could not be used. However, the data used were not acquired at a single moment, and the input data used for the proposed CNN were time series of radar data. More optimal structures for use with larger datasets could be developed based on a transformer network, recurrent neural network or long short-term memory network^44,45^.

Future studies with other populations, such as adults with low fat and high physical activity or obesity and children and adolescents, are warranted to validate the methodology for more varied groups. Additionally, further study is necessary to achieve sufficient accuracy with patients in various postures, such as the supine position, to use the IR-UWB radar as an anthropometric measuring method in clinical practice, since the supine position is most frequently used for clinical procedures. This study was conducted only with subjects standing in the indicated direction for 5 s, and only small movements, such as breathing or changing facial expressions, were allowed. In future work, this technique and the associated module can be implemented with the Internet of Things for various remote health monitoring applications or emergent situations in which a scale cannot be used to measure human weight. Moreover, a real-time version of the radar system will be designed for embedded system implementation to construct a standalone hardware and software solution.

## Limitations

This study has some limitations. The main problem is the dearth of real datasets with anthropometric ground truths for individuals. Second, we used three radar sensors to acquire the signals from various angles. To improve the applicability of the proposed system in the clinical field, an algorithm must be developed that possesses sufficient accuracy with fewer radar sensors.

## Conclusions

For the first time, we demonstrated that an innovative, noncontact, novel, effective method, IR-UWB radar technology integrated with a machine learning algorithm, can measure anthropometric profiles successfully with substantial agreement with BIA in adults. Further studies with younger age groups and better algorithms are warranted before the proposed system can be widely used in clinical practice with the robustness of radar.

## Data Availability

The datasets used and/or analysed during the current study available from the corresponding author on reasonable request.
